# Predictors of Mortality in Pediatric Cardiac Intensive Care Units: A Systematic Review and Exploratory Meta-Analysis

**DOI:** 10.7759/cureus.109337

**Published:** 2026-05-21

**Authors:** Uzoma Ndukwe, Chimaobi Ezekiel Ijioma, Osasumwen Ighodaro

**Affiliations:** 1 Pediatrics, University of Arkansas for Medical Sciences, Little Rock, USA; 2 Pediatrics, Lehigh Valley Health Network, Allentown, USA

**Keywords:** icu, mortality, pcicu, pediatric cardiac intensive care units, predictors, prognostic factor, risk factors

## Abstract

Predictors of mortality in pediatric cardiac intensive care populations remain incompletely characterized. This study aimed to systematically evaluate and synthesize adjusted risk factors across relevant clinical conditions. A synthesis of existing evidence was done to identify the most reliable factors for risk stratification and to guide management in this vulnerable population. A systematic review and meta-analysis were conducted following PRISMA guidelines. A detailed search in PubMed, Embase, EBSCO, and OVID Medline was conducted for studies that were published from January 2020 through March 2026. The search yielded 866 citations. Studies reporting adjusted odds ratios (ORs) or hazard ratios (HRs) for predictors of adverse outcomes in pediatric cardiac intensive care settings were included. Random-effects meta-analysis was performed for predictors reported in at least two studies. Heterogeneity was assessed using the I^2^ statistic. A total of 14 studies, including 60923 patients, met the inclusion criteria. The pooled mortality rate across included studies was 8.98% (95% CI: 4.94-15.78%). A total of 30 predictors were identified. Notable findings, including pooled analyses, demonstrated that extracorporeal membrane oxygenation (ECMO) was associated with increased odds of adverse outcomes (pooled OR 2.26 (1.44-3.55)). Renal replacement therapy showed a strong association (pooled OR 6.7731 (4.3174-10.6255)), albeit with low heterogeneity. Vasoactive therapy and cardiac arrest were also significant predictors, with pooled ORs ranging from approximately 2.7 to 4. Unplanned reoperation demonstrated consistent effects with low heterogeneity, with a pooled OR of 2.3583 (1.4687-3.7867). Key clinical interventions and markers of disease severity, including ECMO, renal replacement therapy, vasoactive therapy, and cardiac arrest, are strongly associated with mortality in pediatric cardiac intensive care populations. These findings may inform risk stratification and clinical decision-making.

## Introduction and background

Pediatric cardiac intensive care has evolved significantly over the past decades, with advances in surgical techniques, perioperative management, and critical care support leading to improved survival among children with congenital and acquired heart disease [1,2]. Despite these improvements, mortality remains substantial, particularly among high-risk populations requiring complex surgical interventions or advanced life-sustaining therapies [3].

Outcomes in pediatric cardiac intensive care units (PCICUs) are influenced by a wide range of factors, including patient-specific characteristics, underlying cardiac pathology, surgical complexity, and postoperative complications. Established risk stratification tools, such as the Society of Thoracic Surgeons-European Association for Cardio-Thoracic Surgery (STAT) mortality categories and the Risk Adjustment for Congenital Heart Surgery (RACHS-1) score, have improved the ability to predict outcomes at a population level [4,5]. However, these models may not fully capture dynamic clinical variables encountered during critical illness, such as organ dysfunction, hemodynamic instability, and the need for advanced supportive therapies.

In clinical practice, predictors of mortality often extend beyond preoperative risk and include markers of acute physiologic deterioration, such as cardiac arrest, renal failure, and escalating vasoactive support. Prior studies have demonstrated that these factors are strongly associated with adverse outcomes in critically ill pediatric populations [6,7]. However, findings across individual studies remain inconsistent due to variations in study design, patient populations, and outcome definitions, limiting generalizability.

Furthermore, the increasing use of advanced interventions, including extracorporeal membrane oxygenation (ECMO) and mechanical circulatory support, has introduced additional complexity in prognostication. While these therapies are life-saving, their association with mortality is often confounded by underlying disease severity. Data from the Extracorporeal Life Support Organization continues to demonstrate substantial mortality rates among pediatric patients requiring extracorporeal support [8].

Given these challenges, there is a need for a comprehensive synthesis of available evidence to identify the most consistent and clinically relevant predictors of mortality in pediatric cardiac intensive care populations. To address these gaps, this systematic review and meta-analysis seek to synthesize the available evidence on predictors of mortality in PCICUs. The specific research question guiding this study is: What clinical, biochemical, intraoperative, and postoperative factors most reliably predict mortality among pediatric patients admitted to cardiac intensive care units?

By consolidating existing knowledge, this study aims to provide clinicians with a clearer understanding of mortality predictors, inform perioperative and postoperative management strategies, and establish benchmarks for evaluating therapeutic interventions. Ultimately, such synthesis has the potential to improve patient safety, guide resource allocation, and enhance survival in this vulnerable population.

Therefore, this systematic review and meta-analysis aimed to evaluate and quantify independent predictors of mortality across studies, assess the consistency of these associations, and determine the overall certainty of evidence using the Grading of Recommendations Assessment, Development and Evaluation (GRADE) framework [9].

## Review

Methods

Study Design

We conducted a systematic review and meta-analysis to identify predictors of mortality among patients admitted to PCICUs. Methods followed the Preferred Reporting Items for Systematic Reviews and Meta-Analyses (PRISMA 2020) guidelines [10]. The protocol was prospectively registered in PROSPERO with registration number CRD420261281673.

Types of Studies

We included observational studies that evaluated predictors or risk factors associated with mortality among pediatric patients in cardiac intensive care settings. Randomized controlled trials, case reports, conference abstracts, editorials, and reviews were excluded.

Population

Studies were considered eligible if they included children aged 0-18 years, including neonates, who were admitted to dedicated PCICUs or mixed PICUs with clearly defined cardiac populations. Eligible populations comprised both postoperative and medical cardiac patients, including those with congenital heart disease (CHD), cardiomyopathy, myocarditis, heart failure, or those requiring advanced support. Studies exclusively evaluating adults, fetal cardiac interventions, or non-ICU populations were excluded.

Exposure/Predictors of Interest

A broad range of variables was considered as potential predictors of mortality, including clinical, demographic, physiological, laboratory, perioperative, and ICU-related factors. These encompassed demographic characteristics (e.g., age, weight), measures of cardiac anatomy and surgical complexity (e.g., RACHS and STAT categories), hemodynamic parameters, markers of organ dysfunction (e.g., lactate levels), therapeutic interventions (e.g., ECMO, mechanical ventilation, and inotropic support), comorbid conditions, and factors related to timing of surgery or postoperative complications. The primary outcome was mortality, defined as PC/ICU mortality, in-hospital mortality, or 30-day postoperative mortality, as reported by individual studies; studies that did not report mortality outcomes were excluded.

Search Strategy

A detailed search of PubMed/MEDLINE, Embase, EBSCO, and OVID Medline was conducted from January 2020 to March 2026 using a combination of controlled vocabulary (Medical Subject Headings (MeSH)/Emtree terms) and keywords related to pediatric cardiac intensive care, CHD, mortality and death, and predictors or prognostic factors, with full electronic search strategies for each database provided in Table 1.

**Table 1:** Electronic Search Strategies Truncation (*) used to capture word variations.

Database	Step		Search Query	Result
PubMed	1		"pediatric"[MeSH] OR pediatric OR children OR neonate OR infant	213
	2		"cardiac intensive care" OR "cardiac ICU" OR "cardiac critical care" OR PCICU OR PICU	
	3		"congenital heart disease"[MeSH] OR CHD OR cardiomyopathy OR myocarditis OR arrhythmia OR heart failure	
	4		mortality OR death	
	5		predictors OR "risk factors" OR "prognostic factors"	
	6		1 AND 2	
	7		3 AND 4	
	8		7 AND 5	
	9		6 AND 8	
Embase	1		pediatrics'/exp OR pediatric*:ti,ab OR child*:ti,ab OR neonat*:ti,ab OR infant*:ti,ab	178
	2		cardiac intensive care'/exp OR 'cardiac icu':ti,ab OR 'cardiac critical care':ti,ab OR pcicu:ti,ab OR picu:ti,ab	
	3		congenital heart disease'/exp OR 'cardiomyopathy'/exp OR 'myocarditis'/exp OR 'arrhythmia'/exp OR 'heart failure'/exp OR chd:ti,ab	
	4		mortality'/exp OR death:ti,ab	
	5		risk factor'/exp OR 'prognosis'/exp OR predictor*:ti,ab OR 'risk factor*':ti,ab OR 'prognostic factor*':ti,ab	
	6		1 AND 2	
	7		3 AND 4	
	8		7 AND 5	
	9		6 AND 8	
EBSCO	1		(MH "Pediatrics+" OR pediatric* OR child* OR neonat* OR infant*)	281
	2		("cardiac intensive care" OR "cardiac ICU" OR "cardiac critical care" OR PCICU OR PICU)	
	3		(MH "Heart Defects, Congenital+" OR MH "Cardiomyopathies+" OR MH "Myocarditis+" OR MH "Arrhythmias, Cardiac+" OR MH "Heart Failure+" OR CHD)	
	4		(MH "Mortality+" OR death)	
	5		(MH "Risk Factors+" OR MH "Prognosis+" OR predictor* OR "risk factor*" OR "prognostic factor*")	
	6		1 AND 2	
	7		3 AND 4	
	8		7 AND 5	
	9		6 AND 8	
OVID	1		exp Pediatrics/OR exp Infant/OR exp Child/OR pediatric*.tw. OR child*.tw. OR neonat*.tw. OR infant*.tw.	194
	2		exp Intensive Care Units, Pediatric/OR (cardiac adj3 (ICU OR intensive care OR critical care)).tw. OR PCICU.tw. OR PICU.tw.	
	3		exp Heart Defects, Congenital/OR exp Cardiomyopathies/OR exp Myocarditis/OR exp Arrhythmias, Cardiac/OR exp Heart Failure/OR CHD.tw.	
	4		exp Mortality/OR death.tw	
	5		exp Risk Factors/OR exp Prognosis/OR predictor*.tw. OR risk factor*.tw. OR prognostic factor*.tw.	
	6		1 AND 2	
	7		3 AND 4	
	8		7 AND 5	
	9		6 AND 8	

Study Selection

**Figure 1:**
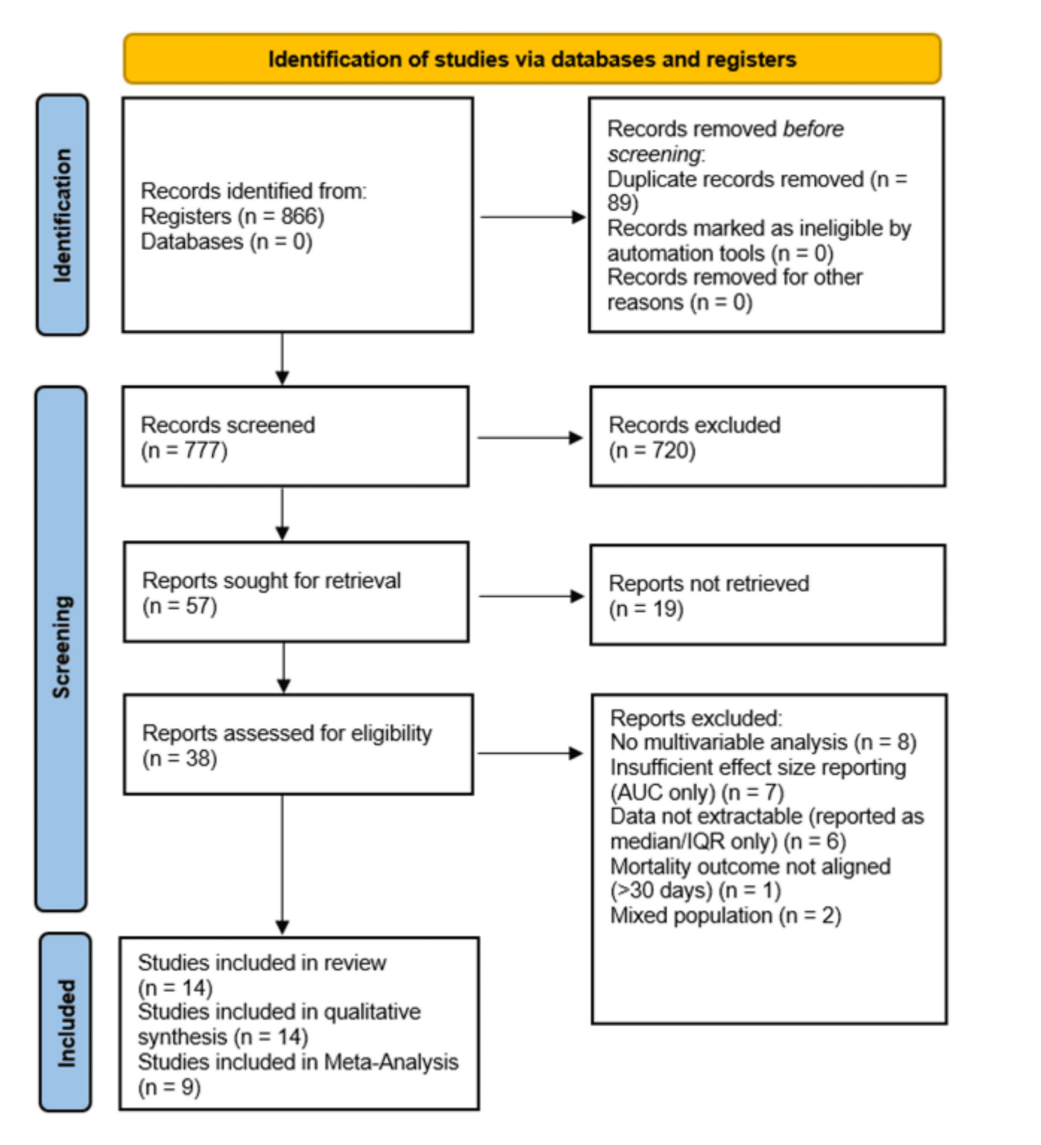
PRISMA Flow Diagram of Study Selection PRISMA: Preferred Reporting Items for Systematic Reviews and Meta-Analyses; AUC: area under the curve

All identified records were imported into reference management software, and duplicates were removed. Two reviewers independently screened the studies in a two-step process, beginning with titles and abstracts, followed by full-text review of articles meeting the inclusion criteria. Any discrepancies were resolved through consensus or by consultation with a third reviewer. The study selection process was summarized using a PRISMA flow diagram as seen in Figure 1.

Data Extraction

Data extraction was performed independently by two reviewers using a standardized, piloted data-extraction form, capturing study characteristics (year, country, design, and setting), population details (sample size, age range, and cardiac diagnoses), ICU type (PCICU vs. PICU), mortality definitions and rates, assessed predictors and statistical methods, effect estimates (adjusted odds ratios (ORs), risk ratios (RRs), or hazard ratios (HRs) with 95% confidence intervals), and variables included in multivariable models. When data were incomplete, the corresponding authors were contacted for clarification.

Multiple effect estimates derived from the same publication were included as independent data points when they represented distinct patient populations or non-overlapping cohorts. For example, DeWitt et al. reported separate analyses (DeWitt 2020a and DeWitt 2020b), which were included independently as they represented different study populations [11].

Risk of Bias Assessment

Risk of bias was assessed using the Newcastle-Ottawa Scale (NOS) for cohort studies [12]. Studies scoring 8-9 stars were considered low risk, 6-7 stars moderate risk, and ≤5 stars high risk. Most included studies were of moderate quality, with several large multicenter studies demonstrating low risk of bias.

Certainty of Evidence Assessment

The certainty of evidence for each identified predictor of mortality was assessed using the GRADE approach [9]. Given that all included studies were observational in design, the certainty of evidence for each outcome was initially rated as low. The certainty was then evaluated across five domains: risk of bias, inconsistency, indirectness, imprecision, and publication bias. Evidence was downgraded by one or two levels for serious or very serious concerns within any domain.

Data Synthesis and Statistical Analysis

Effect measures: Adjusted effect sizes (OR, HR, or RR) were used preferentially. When necessary, effect measures were transformed for comparability.

Meta-analysis: Meta-analyses were performed when ≥2 studies reported the same predictor with sufficiently homogeneous definitions. A random-effects model (DerSimonian-Laird) was used to account for between-study heterogeneity.

Heterogeneity assessment: Statistical heterogeneity was quantified using Tau^2^ (τ^2^) to estimate between-study variance, Cochran’s Q test, and the I^2^ statistic to measure the percentage of variability attributable to heterogeneity.

Publication bias: When ≥2 studies were available, publication bias was to be evaluated using funnel plots and Egger’s regression test.

Software: All analyses were performed in R version 2026.01.1+403 (R Foundation for Statistical Computing, Vienna, Austria) using the metafor, meta, and readxl packages.

Results

A total of 14 studies were included in the systematic review, encompassing a combined sample size of 60,923 pediatric patients across diverse geographic regions, as seen in Table 2. Most studies were conducted in the United States (n=7), followed by China (n=3) and Mexico (n=2), reflecting a predominance of data from high- and middle-income settings. Most studies employed a retrospective observational design (n=14), with only one prospective study identified. Study settings included both multicenter cohorts (n=9) and single-center studies (n=6), with multicenter studies generally contributing larger sample sizes. The study periods spanned from 2005 to 2024, indicating both historical and contemporary data representation. Age ranges included neonates through adolescents, with several studies focusing specifically on infants (<1 year), while others included the full pediatric spectrum (0-18 years). Two analyses from the same study (DeWitt et al., 2020) were included as separate datasets, as they represented distinct patient populations.

**Table 2:** Included Studies R: retrospective; P: prospective; M: multicenter; S: single center DeWitt 2020a and 2020b represent distinct cohorts reported within the same publication and were analyzed separately.

Study ID	First Author	Year	Country	Study Design	Setting	Study Period	Sample Size (N)	Age Range
Peng 2023	Peng et al. [13]	2023	USA	R	M	Aug 2014-Jun 2021	847	2.7-16.0
Morell 2021	Morell et al. [14]	2021	USA	R	M	Aug 2014-Sep 2019	2602	0-18
DeWitt 2020a	DeWitt et al. [11]	2020	USA	R	M	Aug 2014-Dec 2016	2419	0-0.89
DeWitt 2020b	DeWitt et al. [11]	2020	USA	R	M	Aug 2014-Dec 2016	10687	0.09-18
Kamsheh 2025	Kamsheh et al. [15]	2025	USA	R	M	Jan 2010-Dec 2020	10292	0-18
Sanchez-Felix 2026	Sanchez-Felix et al. [16]	2026	Mexico	R	S	Jan 2022-Dec 2024	103	0-4
Sayed 2021	Sayed et al. [17]	2021	USA	R	S	Jan 2012-Aug 2016	102	0-18
Tong 2025	Tong et al. [18]	2025	China	R	M	Jan 2015-Dec 2021	21855	0-18
Zhang 2025	Zhang et al. [19]	2025	China	R	S	May 2019-May 2023	190	0-0.89
Wittenberg 2024	Wittenberg et al. [20]	2024	USA	R	M	Jan 2015-Dec 2020	10966	0-5
Rajapreya 2021	Rajapreya et al. [21]	2021	USA	R	M	Jan 2014-Dec 2017	168	0-18
Gopalakrishnan 2023	Gopalakrishnan et al. [22]	2023	India	R	S	Jan 2020-Dec 2020	437	0-18
Lee 2020	Lee et al. [23]	2020	China	R	M	Jan 2005-Dec 2017	33	0.83-18
Castañuela-Sánchez 2022	Castañuela-Sánchez et al. [24]	2022	Mexico	P	S	Jul 2018-Dec 2019	130	0-18
Sobeih 2020	Sobeih et al. [25]	2020	Egypt	R	S	Jan 2012-Dec 2018	92	0.5-12

As seen in Table 3, across the 15 included studies, mortality rates varied widely depending on underlying diagnosis, illness severity, and ICU type. The pooled mortality rate across included studies was 8.98% (95% CI: 4.94-15.78%). The overall reported mortality ranged from as low as 0.8% (Wittenberg 2024; ventricular septal defect (VSD) closure patients) to as high as 38.7% (Rajapreya 2021; ECMO patients). Studies involving high-risk populations, such as extracorporeal life support and severe myocardial dysfunction, demonstrated the highest mortality rates. For instance, patients requiring ECMO had mortality rates of 38.7% (Rajapreya 2021) and 30.4% (Lee 2020), while cardiomyopathy and post-surgical cohorts also showed elevated mortality (up to 25%). In contrast, studies involving less complex surgical procedures or lower-risk populations reported substantially lower mortality rates, including 1% (Tong 2025; CHD surgery patients) and 0.8% (Wittenberg 2024; VSD closure patients). Among postoperative cardiac surgery populations, mortality varied considerably, ranging from 2% to 24.9%. Most studies defined mortality as in-hospital mortality, although some used ICU mortality or 30-day mortality, contributing to variability in outcome reporting.

**Table 3:** Summary of Study Populations, ICU Settings, and Mortality Outcomes PCICU: pediatric cardiac intensive care unit; CHD: congenital heart disease; CPB: cardiopulmonary bypass; VSD: ventricular septal defect; ECMO: extracorporeal membrane oxygenation

Study ID	Population Description	ICU Type	Mortality Definition	Mortality (n/N)	Mortality Rate (%)
Peng 2023 [13]	Myocarditis	PCICU	In-hospital	53/847	6.3
Morell 2021 [14]	Pulmonary hypertension	PCICU	ICU	260/2602	10
DeWitt 2020a [11]	Post-cardiac surgery	PCICU	ICU	580/2419	24
DeWitt 2020b [11]	Post-cardiac surgery	PCICU	ICU	854/10687	8
Kamsheh 2025 [15]	CHD surgery patients	PICU	In-hospital	2563/10292	24.9
Sanchez-Felix 2026 [16]	CHD surgery patients	PICU	In-hospital	11/103	10.67
Sayed 2021 [17]	Cardiopulmonary bypass	PICU	In-hospital	9/102	8.82
Tong 2025 [18]	CHD surgery patients	PCICU	In-hospital	223/21855	1%
Zhang 2025 [19]	Cardiac surgery with CPB	PICU	In-hospital	22/190	11.60%
Wittenberg 2024 [20]	VSD closure patients	PICU	In-hospital	84/10966	0.80%
Rajapreya 2021 [21]	ECMO patients	PICU	ICU	65/168	38.69%
Gopalakrishnan 2023 [22]	Post-cardiac surgery	PCICU	In-hospital	9/437	2%
Lee 2020 [23]	Fulminant myocarditis on ECMO	PICU	In-hospital	10/33	30.40%
Castañuela-Sánchez 2022 [24]	CHD surgery patients	PCICU	30 days	7/130	5%
Sobeih 2020 [25]	Cardiomyopathy patients	PCICU	In-hospital	23/92	25%

As seen in Table 4, most included studies were of moderate quality (NOS score 6-7), primarily due to single-center design and smaller sample sizes. Several large multicenter cohort studies demonstrated low risk of bias with high methodological quality (NOS score 8-9).

**Table 4:** Risk of Bias Assessment Using the Newcastle-Ottawa Scale

Study ID	Selection (Max. 4)	Comparability (Max. 2)	Outcome (Max. 3)	Total	Risk
Peng 2023 [13]	4	2	3	9	Low
Morell 2021 [14]	4	2	3	9	Low
DeWitt 2020 [11]	4	2	3	9	Low
Kamsheh 2025 [15]	4	2	3	9	Low
Sanchez-Felix 2026 [16]	3	1	2	6	Moderate
Sayed 2021 [17]	3	1	3	7	Moderate
Tong 2025 [18]	4	2	3	9	Low
Zhang 2025 [19]	3	1	2	6	Moderate
Wittenberg 2024 [20]	4	2	3	9	Low
Rajapreya 2021 [21]	3	1	2	6	Moderate
Gopalakrishnan 2023 [22]	3	1	2	6	Moderate
Lee 2020 [23]	3	1	2	6	Moderate
Castañuela-Sánchez 2022 [24]	3	1	2	6	Moderate
Sobeih 2020 [25]	3	1	2	6	Moderate

Across the included studies, multiple independent predictors of mortality were identified, spanning patient characteristics, disease severity, perioperative factors, and critical care interventions, as seen in Table 5.

**Table 5:** Predictors Reported in Single Studies (Not Pooled/Qualitative Synthesis) eGFR: estimated glomerular filtration rate; CPR: cardiopulmonary resuscitation; CPB: cardiopulmonary bypass; PH: pulmonary hypertension; STS: Society of Thoracic Surgeons; AVDSf: alveolar dead space fraction; STAT: Society of Thoracic Surgeons-European Association for Cardio-Thoracic Surgery; RASCH: Risk Adjustment for Congenital Heart Surgery; OR: odds ratio

Predictor	Study ID	Adjusted Effect Size Type	Effect Size (95% CI)	P-value
Congenital heart disease	Peng 2023 [13]	OR	4.1 (1.37-12.16)	0.01
eGFR <30	Peng 2023 [13]	OR	3.6 (1.08-12.27)	0.04
Single-ventricle shunt surgery	Morell 2021 [14]	OR	2.2 (1.1-4.5)	0.022
CPR	Morell 2021 [14]	OR	8.9 (5.6-14.1)	<0.001
Multiple PH therapies	Morell 2021 [14]	OR	2 (1.1-3.6)	0.022
Inhaled nitric oxide use	DeWitt 2020b [11]	OR	1.8 (1.1-2.8)	0.0005
Mechanical circulatory support	DeWitt 2020b [11]	OR	3.9 (2.2-7.1)	<0.001
Any pre-operative STS risk factor	DeWitt 2020b [11]	OR	2.3 (1.2-4.2)	0.0004
AVDSf > 0.25	Sayed 2021 [17]	OR	4.9 (1.45-16.6)	0.0016
STAT categories 2	Tong 2025 [18]	OR	3.29 (2.03-5.33)	<0.001
STAT categories 3	Tong 2025 [18]	OR	5.15 (3.11-8.52)	<0.001
STAT categories 4	Tong 2025 [18]	OR	6.90 (4.41-10.81)	<0.001
Previous cardiac surgery	Tong 2025 [18]	OR	2.09 (1.37-3.21)	0.001
RASCH-2 category ≥ 4	Zhang 2025 [19]	OR	11.93 (1.34-106.10)	0.026
CPB time	Zhang 2025 [19]	OR	1.02 (1.00-1.03)	0.014
Peritoneal hemodialysis	Zhang 2025 [19]	OR	9.252 (1.56-54.96)	0.014
Severe wasting	Wittenberg 2024 [20]	OR	3.38 (1.55-7.35)	0.002
Underweight	Wittenberg 2024 [20]	OR	6.46 (2.81-14.8)	<0.001
Stunting	Wittenberg 2024 [20]	OR	2.73 (1.4-5.34)	0.003
Blood products	Rajapreya 2021 [21]	OR	5.8 (2.7-12.3)	0.048
Sepsis	Gopalakrishnan 2023 [22]	OR	8.6 (1.7-44.9)	0.01
Fluid overload > 5%	Castañuela-Sánchez 2022 [24]	OR	89 (4.3-1813)	<0.001
Hypotension	Sobeih 2020 [25]	OR	9.6 (1.71-54.24)	0.024

Underlying conditions such as CHD were associated with increased mortality (OR 4.1, 95% CI 1.37-12.16, p=0.01). Markers of organ dysfunction, including severely reduced renal function (estimated glomerular filtration rate (eGFR) <30), were also significant predictors (OR 3.6, 95% CI 1.08-12.27, p=0.04). Nutritional status emerged as an important determinant, with underweight (OR 6.46), severe wasting (OR 3.38), and stunting (OR 2.73) significantly increasing mortality.

Higher surgical complexity scores, including STAT mortality categories, demonstrated a stepwise increase in mortality risk. Similarly, higher-risk categories using RACHS-2 score (≥4) were associated with markedly increased mortality (OR 11.93, p=0.026).

Other significant procedural and clinical severity indicators included single-ventricle shunt surgery (OR 2.2), previous cardiac surgery (OR 2.09), and prolonged cardiopulmonary bypass (CPB) time (OR 1.02). Markers of acute critical illness showed some of the strongest associations with mortality, including cardiopulmonary resuscitation (CPR) (OR 8.9, p<0.001), sepsis (OR 8.6, p=0.01), and hypotension (OR 9.6, p=0.024), as well as fluid overload >5% (OR 89, very wide CI) and peritoneal hemodialysis (OR 9.25).

The use of intensive therapies was strongly associated with mortality, including mechanical circulatory support (OR 3.9, p<0.001), inhaled nitric oxide use (OR 1.8, p=0.0005), and multiple pulmonary hypertension therapies (OR 2.0, p=0.022). Additionally, transfusion requirements (blood products, OR 5.8) were associated with increased mortality. Specific physiologic markers such as alveolar dead space fraction (AVDSf) >0.25 were also independently associated with mortality (OR 4.9, p=0.0016), reflecting the importance of cardiac functional parameters.

As seen in Table 6, a meta-analysis of pooled predictors demonstrated several significant associations with mortality in pediatric cardiac intensive care populations. ECMO was significantly associated with increased mortality, with a pooled OR of 2.26 (95% CI 1.44-3.55, p=0.0004) and moderate heterogeneity (I^2^=41.5%), indicating a consistent effect across studies. Vasoactive therapy was also significantly associated with mortality (OR 2.75 (1.26-6.01), p=0.011), although very high heterogeneity (I^2^=99.7%) suggests substantial variability across study populations and clinical contexts. Renal replacement therapy (RRT) demonstrated one of the strongest associations, with a pooled OR of 6.77 (95% CI 4.32-10.63, p<0.0001) and no heterogeneity (I^2^=0%), indicating highly consistent findings. Cardiac arrest was significantly associated with mortality (OR 4.08 (1.83-9.07), p=0.0006), with moderate heterogeneity (I^2^=63.8%). Unplanned surgery was also a significant predictor (OR 2.36 (1.47-3.79), p=0.0004), with moderate heterogeneity (I^2^=45.7%).

**Table 6:** Predictors Reported in Multiple Studies (Pooled/Quantitative Synthesis) A p-value <0.05 was considered statistically significant Int: interpretation; S: significant; NS: non-significant; ECMO: extracorporeal membrane oxygenation; OR: odds ratio; HR: hazard ratio

Predictor	Studies	Effect Measure	Individual Effect Sizes	Pooled Effect (95% CI)	P-value	I^2^ (%)	Int
ECMO	Peng 2023 [13]	OR	2.9 (1.4-6.14)	2.26 (1.44-3.55)	0.0004	41.5	S
	Morell 2021 [14]		3.0 (1.6-5.6)				
	Zang 2025 [19]		1.60 (1.03-2.50)				
Mechanical ventilation	Peng 2023 [13]	OR	22.3 (4.74-104.41)	3.30 (0.97-11.16)	0.055	94.0	NS
	DeWitt 2020a [11]		1.02 (1.01-1.03)				
	DeWitt 2020b [11]		1.01 (1.00-1.02)				
	Tong 2025 [18]		2.476 (1.819-3.371)				
	Sobeih 2020 [25]		14.9 (4.28-51.81)				
Age	Morell 2021 [14]	OR	1.7 (1.1-2.5)	1.27 (0.79-2.04)	0.316	81.7	NS
	Tong 2025 [18]		1.042 (1.03-1.054)				
Vasoactive therapy	Morell 2021 [14]	OR	4.8 (2.6-8.8)	2.75 (1.26-6.01)	0.011	99.7	S
	DeWitt 2020a [11]		1.01 (1-1.02)				
	Kamsheh 2025		3.53 (3.28-3.79)				
	Sobeih 2020		4.52 (1.29-15.81)				
Renal replacement therapy	DeWitt 2020a [11]	OR	8.3 (4.1-16.9)	6.77 (4.32-10.63)	<0.001	0.0	S
	DeWitt 2020b [11]		5.9 (3.3-10.6)				
Cardiac arrest	DeWitt 2020a [11]	OR	5.9 (3.3-10.6)	4.08 (1.83-9.07)	0.0006	63.8	S
	DeWitt 2020b [11]		2.6 (1.2-5.6)				
Unplanned surgery	DeWitt 2020a [11]	OR	3.1 (1.4-6.8)	2.36 (1.47-3.79)	0.0004	45.7	S
	DeWitt 2020b [11]		3.1 (1.7-5.5)				
	Tong 2025 [18]		1.62 (1.02-2.59)				
Lactate	Sanchez-Felix 2026 [16]	HR	2.17 (1.16-4.06)	1.39 (0.67-2.88)	0.375	82.1	NS
	Lee 2020 [23]		1.02 (1.01-1.04)				

Mechanical ventilation showed a large pooled effect (OR 3.30 (0.97-11.16)) but did not reach statistical significance (p=0.055), with very high heterogeneity (I^2^=94%), indicating substantial variability across studies. Age was not significantly associated with mortality (OR 1.27 (0.79-2.04), p=0.316) and demonstrated high heterogeneity (I^2^=81.7%). For the predictor reported as HR, lactate was not significantly associated with mortality (HR 1.39 (0.67-2.88), p=0.375) and showed high heterogeneity (I^2^=82.1%).

**Figure 2:**
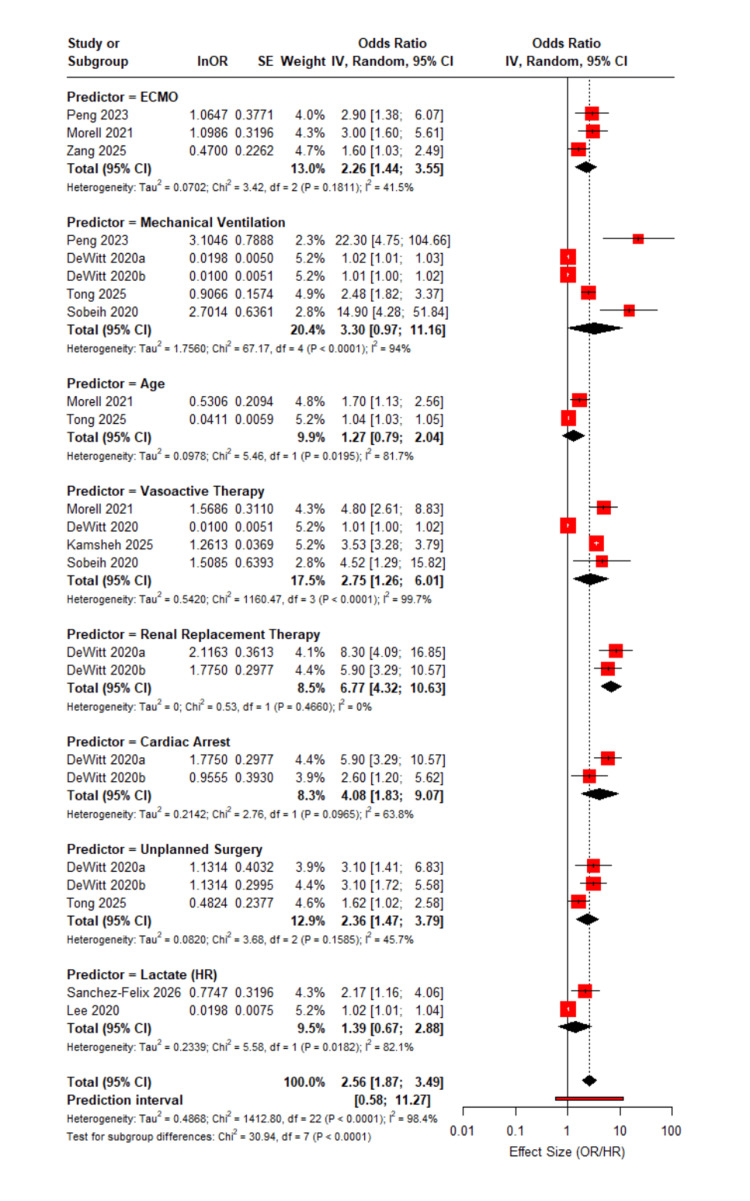
Forest Plot of Pooled Odds Ratios for Mortality Predictors Forest plot showing pooled odds ratios for mortality predictors in pediatric cardiac intensive care units. Subgroup analyses were performed for ECMO, mechanical ventilation, age, vasoactive therapy, renal replacement therapy, cardiac arrest, and unplanned surgery using a random-effects model. A random-effects meta-analysis using the inverse-variance method was performed to pool effect estimates, with heterogeneity assessed using the I^2^ statistic and between-study variance (τ^2^). ECMO: extracorporeal membrane oxygenation Studies included [13-25]

A total of eight multiple predictors were analyzed using a random-effects meta-analysis model as noted in Figure 2. The overall pooled analysis demonstrated a significant association between several clinical variables and mortality in pediatric cardiac intensive care populations.

The overall pooled effect estimate across all predictors was OR 2.56 (95% CI 1.87-3.49), indicating that the presence of identified risk factors was associated with more than a twofold increase in mortality. However, there was considerable heterogeneity (I^2^=98.4%, p<0.0001), suggesting substantial variability across studies and predictors.

There was a statistically significant difference between predictor subgroups (χ^2^=30.94, df=7, p<0.0001), suggesting that the effect of predictors on mortality varies significantly depending on the clinical variable analyzed. The wide prediction interval (0.58-11.27) indicates substantial uncertainty in the expected effect size in future settings, further reflecting the high heterogeneity across studies.

As seen in Table 7, a total of 23 single predictors of mortality were evaluated across observational studies. Overall, the certainty of evidence was low to moderate, primarily due to study design limitations and imprecision. Most predictors, including CHD, eGFR <30, single-ventricle shunt surgery, CPR, multiple pulmonary hypertension therapies, inhaled nitric oxide use, mechanical circulatory support, preoperative Society of Thoracic Surgeons (STS) risk factors, AVDSf >0.25, STAT categories (2-4), previous cardiac surgery, and nutritional indices (severe wasting, underweight, and stunting), demonstrated low certainty of evidence.

**Table 7:** GRADE Certainty for Single-Study Predictors (Qualitative Evidence) All included studies were observational, thus starting at low certainty per the GRADE framework. Imprecision (wide confidence intervals and/or small sample size) led to downgrading across all predictors. No inconsistency was assessed due to single-study evidence per predictor. O: observational; Mod: moderate; GRADE: Grading of Recommendations Assessment, Development and Evaluation; eGFR: estimated glomerular filtration rate; CPR: cardiopulmonary resuscitation; CPB: cardiopulmonary bypass; STS: Society of Thoracic Surgeons; AVDSf: alveolar dead space fraction; STAT: Society of Thoracic Surgeons-European Association for Cardio-Thoracic Surgery; RASCH: Risk Adjustment for Congenital Heart Surgery

Predictor	Study	Study Design	Risk of Bias	Inconsistency		Indirectness	Imprecision	Publication Bias	Overall Certainty (GRADE)
Congenital heart disease	Peng 2023	O	Low	Not applicable	Not serious		Serious	Undetected	Low
eGFR <30	Peng 2023	O	Low	Not applicable	Not serious		Serious	Undetected	Low
Single-ventricle shunt surgery	Morell 2021	O	Low	Not applicable	Not serious		Serious	Undetected	Low
CPR	Morell 2021	O	Low	Not applicable	Not serious		Serious	Undetected	Low
Multiple PH therapies	Morell 2021	O	Low	Not applicable	Not serious		Serious	Undetected	Low
Inhaled nitric oxide use	DeWitt 2020b	O	Low	Not applicable	Not serious		Serious	Undetected	Low
Mechanical circulatory support	DeWitt 2020b	O	Low	Not applicable	Not serious		Serious	Undetected	Low
Any pre-operative STS risk factor	DeWitt 2020b	O	Low	Not applicable	Not serious		Serious	Undetected	Low
AVDSf > 0.25	Sayed 2021	O	Low	Not applicable	Not serious		Serious	Undetected	Low
STAT categories 2	Tong 2025	O	Low	Not applicable	Not serious		Serious	Undetected	Low
STAT categories 3	Tong 2025	O	Low	Not applicable	Not serious		Serious	Undetected	Low
STAT categories 4	Tong 2025	O	Low	Not applicable	Not serious		Serious	Undetected	Low
Previous cardiac surgery	Tong 2025	O	Low	Not applicable	Not serious		Serious	Undetected	Low
RASCH-2 category ≥ 4	Zhang 2025	O	Mod	Not applicable	Not serious		Serious	Undetected	Moderate
Prolonged CPB time	Zhang 2025	O	Mod	Not applicable	Not serious		Serious	Undetected	Moderate
Peritoneal hemodialysis	Zhang 2025	O	Mod	Not applicable	Not serious		Serious	Undetected	Moderate
Severe wasting	Wittenberg 2024	O	Low	Not applicable	Not serious		Serious	Undetected	Low
Underweight	Wittenberg 2024	O	Low	Not applicable	Not serious		Serious	Undetected	Low
Stunting	Wittenberg 2024	O	Low	Not applicable	Not serious		Serious	Undetected	Low
Blood products	Rajapreya 2021	O	Mod	Not applicable	Not serious		Serious	Undetected	Moderate
Sepsis	Gopalakrishnan 2023	O	Mod	Not applicable	Not serious		Serious	Undetected	Moderate
Fluid overload > 5%	Castañuela-Sánchez 2022	O	Mod	Not applicable	Not serious		Serious	Undetected	Moderate
Hypotension	Sobeih 2020	O	Mod	Not applicable	Not serious		Serious	Undetected	Moderate

Predictors such as RACHS-2 category ≥4, prolonged CPB time, peritoneal dialysis, blood product transfusion, sepsis, fluid overload >5%, and hypotension were graded as having moderate certainty of evidence, reflecting a higher risk of bias but consistent findings across studies without concerns for inconsistency or indirectness.

Across all predictors, imprecision was the most common reason for downgrading, due to wide confidence intervals and limited sample sizes. No significant inconsistency or publication bias was identified.

As seen in Table 8, the certainty of evidence ranged from very low to moderate, with the highest certainty observed for unplanned reoperation (moderate), supported by relatively consistent findings across studies and acceptable precision. In contrast, most other predictors were graded as low certainty, including ECMO, RRT, and cardiac arrest, primarily due to imprecision and moderate heterogeneity. Predictors such as mechanical ventilation and vasoactive therapy were assigned very low certainty, reflecting substantial heterogeneity (I^2^>90%) and wide confidence intervals, which limit confidence in the pooled estimates. Similarly, lactate was graded as very low certainty due to reliance on a small number of studies and inconsistent findings. Although age was not significantly associated with mortality, the certainty of evidence was rated as low, largely due to heterogeneity across studies and variability in age definitions.

**Table 8:** GRADE Certainty of Evidence for Pooled Predictors (Quantitative Evidence) All studies were observational, starting at low certainty per the GRADE methodology. Inconsistency (heterogeneity across studies) and imprecision (wide confidence intervals/small sample sizes) were the primary reasons for downgrading. GRADE: Grading of Recommendations Assessment, Development and Evaluation; ECMO: extracorporeal membrane oxygenation

Predictor	No. of Studies	Study Design	Risk of Bias	Inconsistency	Indirectness	Imprecision	Publication Bias	Overall Certainty (GRADE)
ECMO	3	O	Low	Moderate	Not serious	Serious	Undetected	Low
Mechanical ventilation	5	O	Low	Serious	Not serious	Serious	Undetected	Very Low
Age	2	O	Low	Serious	Not serious	Moderate	Undetected	Low
Vasoactive therapy	3	O	Low	Serious	Not serious	Serious	Undetected	Very Low
Renal replacement therapy (RRT)	2	O	Low	Low	Not serious	Moderate	Undetected	Low
Cardiac arrest	2	O	Low	Moderate	Not serious	Moderate	Undetected	Low
Unplanned reoperation	3	O	Low	Moderate	Not serious	Moderate	Undetected	Moderate
Lactate	2	O	Moderate	Moderate	Not serious	Moderate	Undetected	Very Low

Discussion

This systematic review highlights several important features of the existing literature on pediatric cardiac intensive care outcomes. The evidence base is overwhelmingly composed of retrospective observational studies, which inherently limit the ability to establish causality and introduce potential biases, including selection bias and residual confounding. This is consistent with the clinical and ethical challenges of conducting randomized studies in critically ill pediatric populations. There is a notable predominance of studies from high-resource settings, particularly the United States and China. While this strengthens internal validity due to robust data systems and large registries, it may limit generalizability to low-resource settings, where disease burden, access to advanced therapies, and outcomes may differ significantly. The inclusion of both multicenter and single-center studies introduces variability in clinical practice patterns, resource availability, and patient populations. Multicenter studies contributed to most patients and likely provide more general estimates, whereas single-center studies, though smaller, may offer more granular clinical insights. The wide variation in sample sizes reflects differences in study design and scope. Larger registry-based studies enhance statistical power but may lack detailed clinical variables, while smaller studies may provide richer clinical detail at the expense of external validity. Inclusion of multiple cohorts from the same study may introduce clustering; however, these analyses represented distinct populations and were therefore treated independently.

The wide variation in mortality rates observed across studies reflects the heterogeneous nature of pediatric cardiac intensive care populations, particularly with respect to disease severity and treatment intensity. Patients requiring advanced life support, such as ECMO, consistently demonstrated the highest mortality rates, highlighting the severity of illness in this subgroup. Despite advances in extracorporeal support, mortality remains high, emphasizing the need for improved patient selection, timing of initiation, and post-ECMO management strategies. Similarly, elevated mortality in patients with cardiomyopathy and fulminant myocarditis underscores the critical nature of myocardial dysfunction in pediatric populations and the challenges associated with its management [26]. In contrast, markedly lower mortality rates in cohorts undergoing elective or lower-risk cardiac procedures, such as VSD closure, likely reflect both advances in surgical techniques and improvements in perioperative care, particularly in high-volume centers [27].

The variability observed among postoperative cardiac surgery studies (ranging from 2% to nearly 25%) suggests that outcomes are influenced by multiple factors, including case complexity (e.g., simple vs. complex CHD), institutional expertise, availability of advanced support, and patient comorbidities. Additionally, the predominance of data from specialized centers (PCICU/PICU) may underestimate mortality in lower-resource settings, where access to advanced interventions is limited.

This analysis highlights that mortality in pediatric cardiac intensive care is driven by a multifactorial interplay of baseline patient risk, surgical complexity, and acute critical illness. Underlying disease burden and comorbidities, such as CHD and renal dysfunction, significantly increase mortality risk. Prior studies have demonstrated that preoperative comorbidities and end-organ dysfunction are strong predictors of adverse outcomes in pediatric cardiac populations [1].

CPR was a potent predictor, reflecting the critical severity of patients requiring resuscitative efforts. Similarly, the need for mechanical circulatory support and multiple pulmonary hypertension therapies highlights the role of advanced support requirements as markers of hemodynamic instability and refractory disease. The use of inhaled nitric oxide further suggests that severe pulmonary vascular dysfunction contributes significantly to adverse outcomes. In addition, echocardiographic markers such as elevated AVDSf indicate that impaired ventricular function is a critical determinant of prognosis.

Surgical complexity scores, including the STAT mortality categories and RACHS-1 score, demonstrated a clear dose-response relationship with mortality in this review. These scoring systems have been extensively validated and remain central to risk stratification and benchmarking in congenital heart surgery [28].

Markers of acute physiological instability, such as hypotension and sepsis, were among the strongest predictors of mortality. This is consistent with prior literature showing that hemodynamic collapse and systemic inflammatory states are associated with significantly increased mortality in critically ill pediatric patients [29]. Early recognition and timely intervention remain crucial in improving outcomes.

The markedly elevated OR associated with fluid overload (>5%) aligns with growing evidence linking fluid accumulation to worse outcomes in pediatric critical care. Fluid overload has been associated with impaired oxygenation, prolonged mechanical ventilation, and increased mortality, particularly in cardiac populations [30].

Similarly, the association between advanced therapies, such as mechanical circulatory support and inhaled nitric oxide, and mortality likely reflects confounding by indication, as these interventions are typically reserved for the most severely ill patients. Prior studies in pediatric ECMO and critical care populations have consistently demonstrated high mortality rates despite technological advances [31].

Nutritional status also emerged as a significant predictor, with underweight, wasting, and stunting associated with increased mortality. Malnutrition has been widely recognized as an independent risk factor for poor surgical and critical care outcomes, contributing to impaired immune function, delayed recovery, and increased susceptibility to complications [32]. Procedural factors such as prolonged CPB time and prior cardiac surgery further emphasize the role of operative complexity and perioperative management in determining outcomes.

Among the most robust findings, RRT demonstrated a strong and consistent association with mortality, with no observed heterogeneity. This suggests that renal dysfunction is a reliable and reproducible marker of poor prognosis, likely reflecting multi-organ failure and advanced critical illness. Similar findings have been reported in pediatric critical care literature, where acute kidney injury and the need for dialysis are strongly linked to increased mortality [7].

Cardiac arrest was also significantly associated with mortality, reinforcing the well-established relationship between hemodynamic collapse and poor outcomes. This finding underscores the importance of early recognition and intervention to prevent progression to arrest [33].

ECMO was associated with a twofold increase in mortality risk. While this may suggest a strong association, it is important to recognize the role of confounding by indication, as ECMO is typically reserved for the most critically ill patients. Prior studies from the Extracorporeal Life Support Organization have similarly reported high mortality rates despite advances in extracorporeal support [8].

The association between vasoactive therapy and mortality, despite statistical significance, was accompanied by extremely high heterogeneity. This likely reflects variability in: indications for vasoactive support, differences in dosing and scoring systems (e.g., vasoactive-inotropic score (VIS)), and underlying patient populations. Thus, while vasoactive therapy is a marker of severity, its pooled estimate should be interpreted cautiously.

Unplanned surgery demonstrated a consistent and significant association with mortality, highlighting the impact of postoperative complications and surgical failure on patient outcomes. This finding aligns with previous literature emphasizing the importance of surgical quality and perioperative management [1].

In contrast, mechanical ventilation, despite a large effect size, did not reach statistical significance due to wide confidence intervals and substantial heterogeneity. This suggests that ventilation alone may not independently predict mortality but rather reflects underlying disease severity and variability in clinical practice. Similarly, age was not a significant predictor, likely due to heterogeneity across studies, including differences in age categorization and population characteristics.

The lack of significance for lactate in the pooled analysis contrasts with prior studies demonstrating its prognostic value. This discrepancy is likely due to limited sample size and heterogeneity in measurement timing and thresholds. Overall, the most consistent predictors of mortality were those reflecting multi-organ dysfunction and acute physiologic collapse, whereas variability in clinical practices and patient populations contributed to heterogeneity in other predictors.

The overall risk of bias was moderate to serious, and the certainty of evidence was generally low due to reliance on observational data, heterogeneity across studies, and imprecision of effect estimates. Despite limited numbers, this study demonstrates methodological rigor, including consistent direction across studies, low-to-moderate heterogeneity, and inclusion of clinically relevant pediatric ICU populations.

Limitations

This analysis is limited by the small number of studies included, which restricts the precision and generalizability of pooled estimates. Several predictors were identified in single studies and could not be pooled, highlighting the need for further multicenter studies to validate these associations. The inclusion of studies whose study period spans nearly two decades introduces potential temporal bias, as advances in surgical techniques, critical care management, and supportive therapies (e.g., ECMO) may have significantly influenced outcomes over time. Overall, the certainty of evidence was limited by the observational design of included studies, the large number of full-text articles that could not be retrieved, inconsistency across results, and imprecision in effect estimates.

## Conclusions

This systematic review and meta-analysis identified several key predictors of mortality in pediatric cardiac intensive care populations, with the most consistent associations observed for markers of multi-organ dysfunction and severe physiologic instability. The findings reveal that mortality risk in this population is multifactorial and cumulative, reflecting the combined effects of baseline patient characteristics, surgical complexity, and the progression of critical illness. While established risk stratification tools such as the STAT mortality categories and RACHS-1 score remain valuable, this study underscores the importance of incorporating dynamic clinical variables into risk assessment models. Our limitations highlight the need for standardized reporting and prospective multicenter studies to improve the robustness and generalizability of findings. Clinically, early identification of high-risk patients and timely intervention targeting modifiable factors such as fluid management, hemodynamic support, and prevention of organ dysfunction may improve outcomes. Future research should focus on developing integrated predictive models that combine baseline risk factors with real-time clinical parameters to enhance prognostication and guide management in pediatric cardiac intensive care.
